# 

**Published:** 1888-06

**Authors:** 


					﻿JUKTK, 1888,
OLD SERIES
Vol. xxv
NEW SERIES
Vol. V.—No 4.\
& 1855 0




^TXtA^Ee>*c/

W.F. WESTMOREL AND. M ol
H.V.M,MILLER. M.D.LL .D*
published By james rharrison&ege
iff	Atlanta,ga. Jg
SUBSCRIPTIONS 12.60 A YEAR.
				

